# Neutrophil Extracellular Traps Do Not Induce Injury and Inflammation in Well-Differentiated RSV-Infected Airway Epithelium

**DOI:** 10.3390/cells11050785

**Published:** 2022-02-24

**Authors:** Rosalie S. N. Linssen, Adithya Sridhar, Giulia Moreni, Nicole N. van der Wel, Job B. M. van Woensel, Katja C. Wolthers, Reinout A. Bem

**Affiliations:** 1 Pediatric Intensive Care Unit, Emma Children’s Hospital, Amsterdam UMC, Location AMC, 1105 AZ Amsterdam, The Netherlands; j.b.vanwoensel@amsterdamumc.nl; 2 OrganoVIR Lab, Department of Medical Microbiology, Amsterdam UMC, Location AMC, University of Amsterdam, 1100 AZ Amsterdam, The Netherlands; a.sridhar@amsterdamumc.nl (A.S.); g.moreni@amsterdamumc.nl (G.M.); k.c.wolthers@amsterdamumc.nl (K.C.W.); 3 Electron Microscopy Center Amsterdam, Department of Medical Biology, Amsterdam UMC, Location AMC, University of Amsterdam, 1105 AZ Amsterdam, The Netherlands; n.n.vanderwel@amsterdamumc.nl

**Keywords:** neutrophils, neutrophil extracellular traps, infection, RSV, respiratory syncytial virus, cell culture, human airway epithelial

## Abstract

Respiratory syncytial virus (RSV) lower respiratory tract infection (LRTI) causes a major burden of disease. The host response in RSV-LRTI is characterized by airway epithelial injury, inflammation and neutrophil influx, with the formation of neutrophil extracellular traps (NETs). However, the precise role of NETs in the pathophysiology of RSV-LRTI remains to be elucidated. Here, we used well-differentiated human airway epithelial cultures (HAE) of a pediatric and adult donor to study whether NETs cause airway epithelial injury and inflammation in the setting of RSV infection. The exposure of uninfected and RSV-infected HAE cultures to NETs, as produced by stimulation of neutrophils by a low dose of phorbol 12-myristate 13-acetate (PMA), did not induce or aggravate cell injury or inflammation. RSV infection of HAE cultures caused release of pro-inflammatory cytokines such as IL-6 and RANTES in both adult and pediatric cultures, but the differential gene expression for regulated cell death differed between culture donors. In this in vitro airway epithelial model, NETs in the setting of RSV infection did not cause or aggravate epithelial injury or inflammation.

## 1. Introduction

Lower respiratory tract infection (LRTI) due to the respiratory syncytial virus (RSV) causes a major burden of disease in infants as well as the elderly worldwide [1,2,3]. The pathophysiology of RSV-LRTI is characterized by small airway inflammation, desquamation of infected and dead epithelial cells (i.e., cell sloughing), impaired mucociliary clearance and massive recruitment of neutrophils [4,5,6,7,8]. These neutrophils are involved in RSV clearance via phagocytosis, the release of neutrophilic granule content and the formation of neutrophil extracellular traps (NETs) [9,10,11,12]. NETs consist of a chromatin fiber network studded with the constituents of azurophilic granules, including neutrophil-elastase (NE), myeloperoxidase and citrullinated histones (CitH3) [13]. The presence of NETs in the airways in RSV-LRTI has been demonstrated [11,14], but the precise role of NETs in the setting of RSV-LRTI remains unclear. 

In vitro airway epithelial monolayer studies have previously shown that NETs are cytotoxic, induce epithelial cell death and cause a pro-inflammatory response via IL-6 and CXCL8 (IL-8) release [15,16]. In addition, in one study the exposure of RSV-infected Hep2 cells to different NET concentrations was associated with a dose-dependent decrease of syncytia formation in cells when using a low multiplicity of RSV infection (MOI 0.5), but not for higher MOI [17]. However, in this study the interpretation of the results is somewhat clouded by the detachment of cells exposed to high concentrations of NETs [17]. A recent study, using well-differentiated human airway epithelium (HAE) cultures, showed that NETs lead to an inflammatory response via increased IL-8 and IL-1α release [18]. 

However, until now limited knowledge exists about the effects of NETs in the context of RSV-infected HAE [19,20]. As airway ciliated cells are considered to be the primary site for RSV infection and replication [21,22], the use of such HAE cultures to model the human airways is preferred over conventional monolayer cell cultures [23]. Furthermore, the use of HAE cultures enables the comparison between both pediatric and adult donors, establishing a much-needed age-relevant human airway model [23,24]. Therefore, in this study, we aimed to determine the age-related cytotoxic and pro-inflammatory responses of RSV-infected bronchial HAE cultures to NETs. 

## 2. Materials and Methods

### 2.1. HAE Cultures

Primary cells for bronchial airway cultures were purchased from Epithelix (Sàrl, Geneva, Switzerland). These cells originated from a pediatric (two-year-old Caucasian female, Epithelix 02AB066001) and an adult donor (62-year-old, non-smoking, Hispanic male, Epithelix 02AB079301). Cells were seeded in a T75 flask with StemCell PneumaCult™-Ex Plus basal expansion medium (Catalog #05040 STEMCELL Technologies, Cambridge, UK, supplements added according to the manufacturer’s instruction, 1% penicillin/streptomycin, Lonza). After reaching 80% confluence, cells were trypsinized (Lonza) and seeded on rat-tail collagen coated inserts (collagen ENZO ALX 522-435-0020, 250× diluted in 0.1% acetic acid, 6.5 mm insert, 0.4 µm, PET European article number 734-3263, VWR) at a cell density of approximately 80,000 cells per insert or passaged with a density of 750,000 cells per flask. Cells were used up to maximum of four passages. Cell cultures were kept submerged for three to four days in expansion media with 10 µM Rho Kinase (ROCK) inhibitor (Millipore via Sigma-Aldrich, Darmstadt, Germany, product number SCM075). Subsequently, differentiation was initiated by removing the apical medium and changing the basolateral cell culture medium to PneumaCult™-ALI differentiation medium (StemCell PneumaCult™-ALI Medium, Catalog #05001, STEMCELL Technologies, Cambridge, UK, supplements added according to manufacturer’s instruction, 1% penicillin/streptomycin). Culture medium in the basolateral compartment was refreshed every 2–3 days (550 µL). HAE cultures were maintained up to 25–28 days in differentiation medium before use. Transepithelial electrical resistance (TEER) was measured using an EVOHM TEER device every week [25]. Before use, monolayer quality was ensured through visualization of cilia beating and TEER measurements > 200 Ω/cm^2^ to confirm tight junction integrity.

### 2.2. Immunostaining of HAE Cultures

Inserts were fixed in 4% paraformaldehyde, washed with PBS and then the fixed membranes were removed from the inserts. Cells were permeabilized with 0.1% Triton in PBS for 15 min and then blocked overnight with 10% BSA–0.5% Tween 20 (both: Sigma-Aldrich, St. Louis, MO, USA) in PBS to reduce binding of unspecific antibodies. Subsequently, the inserts were washed with PBS 0.5%–Tween 20 after which the primary antibodies (diluted in 3% BSA–0.5% Tween 20 in PBS) were added and incubated overnight. The next day inserts were washed in PBS 0.5% Tween and secondary antibodies (diluted in 3% BSA and 0.5% Tween) were added. Cells were stained with DAPI (cell nuclei), ciliated cells (anti-b tubulin 1:1000, recombinant with Alexa Fluor 647 Abcam Catalog ab204034), goblet cells (anti-MUC5AC, 1:50, recombinant with Alexa Fluor 555 Abcam Catalog ab218714) and for RSV (primary antibody goat anti- RSV 1:100, Abcam Catalog ab20745, with secondary donkey anti-goat Alexa Fluor 488, 1:1000, Abcam Catalog ab150129). Finally, inserts were washed and mounted on glass slides.

### 2.3. Neutrophil Isolation and Stimulation

Retrieval of NET supernatants and supernatant from unstimulated neutrophils was performed as previously described [26]. In short, neutrophils were collected from human blood from adult donors, from buffy coats provided by Sanquin Blood Bank (Amsterdam, The Netherlands). Neutrophils were isolated using density separation and centrifugation (Lymfoprep^TM^; StemCell Technologies, Vancouver, BC, Canada) with subsequent erytrolysis (ammonium chloride potassium, ACK lysis buffer, Quality Biological). Neutrophils were cultured in RPMI 1640 media supplemented with 2% fetal calf serum for 3.5 h at 37 °C in the presence of 50 nM phorbol 12-myristate 13-acetate (PMA) to induce NETosis. At this concentration PMA induces NETosis rather than cell death [15]. For control experiments, neutrophils were cultured in the absence of PMA (unstimulated neutrophils). Neutrophils were seeded at a density of 30 million cells in a 10 mm dish (confluent but no overgrowth to avoid cell stress due to over seeding). After 3.5 h, the culture media (with or without PMA) was removed. Cultures were washed three times with PBS followed by repeated pipetting with 2 mL of PBS to loosen the NETs. The material was transferred to tubes and centrifuged to pellet down remaining neutrophils. The supernatant from stimulated (NETs) and unstimulated neutrophils (control) was collected and stored at −80 °C. NET concentration was quantified indirectly using a Quant-iT PicoGreen assay to quantify extracellular dsDNA (Thermo Fisher, Catalog P7589, Thermo Fisher Scientific, Hillsboro, OR, USA, used according to the manufacturer’s instructions). 

### 2.4. NET Visualization

Neutrophils were seeded on coverslips at a density of 100,000 cells/coverslip and treated as described above. The NETs or the unstimulated neutrophils were fixed using 4% paraformaldehyde. NETs were visualized through co-localization of extracellular DNA (DAPI), neutrophil elastase (Neutrophil Elastase with Alexa Fluor 594, Catalog SC-53388AF594, Santa Cruz Biotechnology) and citrullinated Histone H3 (rabbit anti-human citrullinated anti-histone H3, Abcam Catalog AB5103 with donkey anti-rabbit Alexa 647, Thermo Fisher, Catalog P36931) using confocal microscopy as previously described [11,26]. 

### 2.5. RSV Infection

RSV-A2 was received from Prof. L. Bont, UMC Utrecht. RSV-A2 was cultured in Hep2 cells (p19 and p20) and harvested at a final virus titer of 4.8 tissue culture infectious dose (TCID50)/mL, as calculated using the Reed and Meunch method. On the day of infection, cultures were washed three times with 200 µL PBS to remove any excess mucus. Then, 50 µL of RSV (MOI = 0.005) was added to the apical side of the inserts and incubated at 37 °C, 5% CO2 for two hours. RSV suspension was removed, and cultures were washed three times with PBS to remove excess RSV. An amount of 100 µL PneumaCult^TM^-ALI medium was added to the apical side and incubated at 37 °C with 5% CO_2_ for 45 min (infection day 0). After incubation, apical medium was collected as the initial time point of infection. The cultures were then sampled apically as specified every day up to six days. 

### 2.6. Quantitative Real-Time Polymerase Chain Reaction (RT-qPCR) for RSV Infection

To confirm RSV replication in the cell cultures, we performed RT-qPCR. Viral RNA was isolated from the collected apical medium samples using Bioline isolate II RNA mini kit (BIO-52073, Meridian Bioscience^®^, Cincinnati, OH, USA), as per manufacturer’s instructions. Eluted RNA (40 µL) was reverse transcribed as described earlier [27]. Then, 5 µL of the reverse transcribed complementary DNA was used for RT-qPCR with the LightCycler^®^ 480 Probes Master on the CFX connect Real-Time PCR Detection System (Bio-Rad, Hercules, CA, USA). Cq values were converted into viral copy numbers using a standard curve based on known concentrations of the RSV genome. The following primer sets (Biolegio, Nijmegen, The Netherlands) were used for the RT-qPCR. Forward primer: ATG-AAC-AGT-TTA-ACA-TTA-CCA-AGT; reverse primer: GTT-TTG-CCA-TAG-CAT-GAC-AC, and Probe: 6FAM-TGA-CTT-CAA AAA-CAG-ATG-TAA-GCA-GCT-CC-BBQ.XX. 

### 2.7. HAE Culture Exposure to NETs or Unstimulated Neutrophil Supernatant

HAE cultures were carefully washed three times with 100 µL PneumaCult™-ALI Maintenance medium. HAE cultures were exposed to NET supernatant, supernatant of unstimulated neutrophils, or PBS by apically adding 50 µL of supernatant to the HAE cultures and incubating at 37 °C with 5% CO_2_ for 24 h. After 24 h, another 100 uL of Pneumacult medium was added to the apical insert side, after which the total volume of 150 uL was collected for experimental read-outs. For RSV-infected HAE cultures, inserts were infected with RSV 48 h prior to exposure to NET supernatant, unstimulated neutrophil supernatant, or PBS. All tests were performed in triplicate from two separate experiments per HAE donor.

### 2.8. LDH Cytotoxicity Assay

Cytotoxicity was determined by a Lactate dehydrogenase (LDH) cytotoxicity assay (CyQUANT^TM^, Invitrogen, Catalog C20300, Thermo Fisher Scientific, Oregon, USA). LDH, a cytosolic enzyme, is released by cells upon damage of the plasma membrane. Samples were diluted five times in cell culture medium before LDH measurements. The percentage of cytotoxicity (%) in each culture was calculated using the value corresponding with maximal cytotoxicity when lysing the cells (lysing as provided in the CyQUANT™ assay) as a reference (denominator). 

### 2.9. Scanning Electron Microscopy of Cell Cultures

For scanning electron microscopy, samples were fixed with McDowell phosphate buffer. Samples were consequently dehydrated in a series of increasing ethanol concentrations and submerged in a small amount of hexamethyldisilazane (HMDS) for at least to 30 min. Samples were allowed to air-dry by evaporation. Before imaging, samples were sputter coated with 4 nm of platinum/palladium using a Leica EM ACE600 and then imaged at 3 kV using a Zeiss Sigma, Jena, Germany 300 FESEM.

### 2.10. Measurement of Cytokine Release

Cytokine response was measured with a customized 5-plex Luminex^®^ assay (ProcartaPlex, Invitrogen Thermo Fisher Scientific, Waltham, MA, USA). Cytokines included in the panel were: IL-6 as a well-established pro-inflammatory cytokine [28,29], IL-8 (CXC8) as a marker for inflammation and as neutrophil attractant [16,28], IL-9 which has been associated with the upregulation of mucin expression and remodeling of the airways [30,31], IL-29 (IFN lambda 1) as a marker for the infection of ciliated cells [29,32], and RANTES (CCL-5), a chemoattractant expressed by airway epithelium in response to viral infections [29,33,34,35]. For Luminex, the samples collected from three inserts were pooled. 

### 2.11. mRNA-Related Gene Expression (NanoString)

We performed mRNA-related gene expression analysis via a NanoString analysis with a customized NanoString code set (nCounter, NanoString Technologies, Seattle, WA, USA). This custom code set included all gene sequences from the pre-designed commercially available NanoString apoptosis panel and MYD88 and RIPK1 (probes according to NanoString necrosis panel). Five reference genes were included and used for the normalization of data: HPRT1, ABCF1, GUSB, RPL0 and LDHA. The full list of genes is presented in Supplementary Methods 1. For NanoString analysis, RNA was isolated from the cells (Bioline Isolate II RNA Mini Kit). Quality and concentration of the RNA eluates was determined with automated electrophoreses via RNA screen tape analysis (TapeStation, Agilent, Santa Clara, CA, USA). Only samples with RNA integrity number equivalent (RINe) values ≥7.5 were used for further analysis. RNA samples were diluted in RNA-free water to a final dilution of 10 ng/uL. Samples were processed according the NanoString hybridization protocol (65 °C and 12 h long according to Gene Expression RNA CodeSet Hybridization Protocol MAN-10056) and then added to NanoString cartridges up to 50 ng of total RNA. NanoString nSprint analysis was performed according to standard NanoString procedures. 

## 3. Statistical Analysis 

Statistical analysis was performed using Graphpad Prism 9.1.0. We used one-way ANOVA and post hoc Dunnett’s tests to check for differences between treatment groups compared to controls. Data are presented as means with standard deviations; a *p* value < 0.05 was considered statistically significant. 

### Analysis of NanoString Data

Statistical comparisons for the gene expression were carried out with NanoString nSolver Advanced Analysis Software 4.0. The NanoString software determines the gene expression for individual genes using the probe counts for each gene, after normalization against the reference genes. For differential gene expression, the gene expression in the samples is compared against a threshold of 10-fold the background noise. We first detected overall upregulation for genes associated with signaling pathways (pathway scores) as provided by NanoString. The NanoString nSolver software automatically calculates the pathway score using a standardized method, based on a previously described singular value decomposition algorithm [36]. For the pathway scores, the data from multiple genes that belong to a specific pathway are summarized into a single score (nCounter Advanced Analysis Software manual MAN-10030-03). The list of genes related to the pathway’s immune system and regulated cell death are presented in Supplemental Table S1. Next, we studied the differential expression of individual genes as provided via the nSolver Advanced Analysis software (nCounter Advanced Analysis Software manual MAN-10030-03, page 50). Adjusted *p*-values for differential expression outcomes were calculated by the nCounter software using a Benjamini–Yekutieli False Discovery Rate (FDR). We considered a gene significantly up- or downregulated when the adjusted *p* < 0.05.

## 4. Results

### 4.1. RSV-A Successfully Infected Ciliated Cells in HAE Cultures

**Figure 1:**
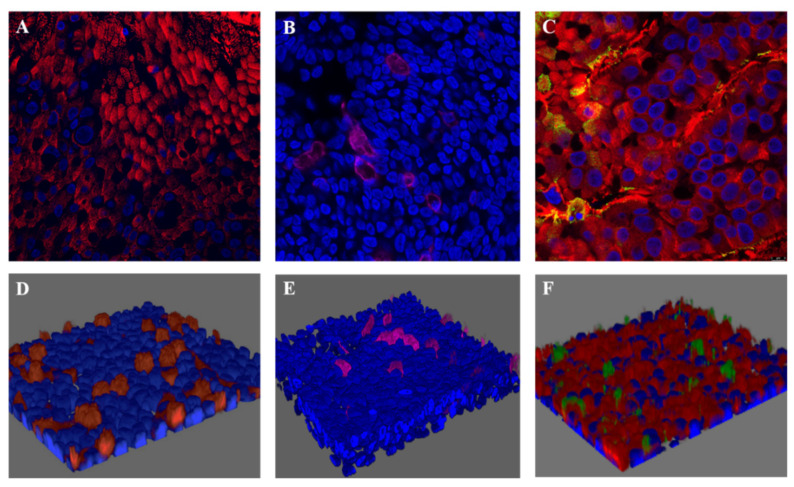
Confocal images and 3D reconstruction of uninfected and RSV-infected HAE cultures. Legend: Immunofluorescence confocal microscopy images of an uninfected adult HAE culture stained for ciliated (**A**) and goblet cells (**B**). Confocal image of an RSV-infected adult HAE culture after 120 h of RSV infection (**C**). The 3D illustrations of an uninfected HAE culture (**D**,**E**); 3D illustration of an RSV-infected HAE culture (**F**). Blue: cell nuclei (DAPI staining), red: ciliated cells (staining with anti-b tubulin antibody), magenta: goblet cells (staining with anti-MUC5AC), green: RSV infection, primarily visible in ciliated cells (staining with polyclonal anti-RSV antibodies).

**Figure 2:**
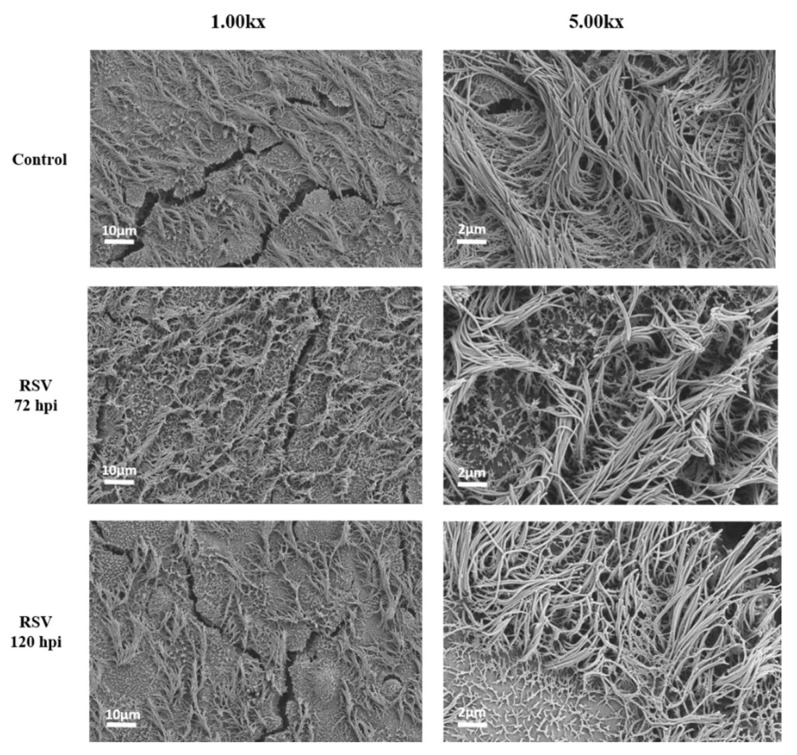
Scanning electron microscopy (SEM) of uninfected and RSV-infected HAE cultures at 72 and 120 h post infection (hpi). SEM pictures show a patchy pattern of disorganized ciliated cells and areas with loss of ciliated cells in the RSV-infected cultures as compared to controls. SEM pictures at 1.00 k× and 5.00 k× magnification (Zeiss). HAE cultures were derived from the adult donor. Note: systematic comparisons for the areas with cell damage between uninfected and RSV-infected HAE at 72 and 120 hpi were not performed.

To create an age-relevant in vitro airway epithelium model that allowed us to study the effects of NETs in the context of RSV-LRTI, we first established well-differentiated HAE cultures from bronchial primary cells from an adult and pediatric donor. Cilia beating became visible between 14–21 day after air-lifting the cultures on day zero. We confirmed that HAE cultures displayed pseudostratified airway epithelium with ciliated and goblet cells (Figure 1). Quality of the HAE cultures was confirmed by the observation of cilia beating via light microscopy and TEER > 200 ohm/cm^2^. HAE cultures were exposed at the apical site to RSV-A2. Active infection of the ciliated cells was confirmed by immunofluorescence (Figure 1) and viral replication over time was confirmed by RT-qPCR (Supplemental Figure S1). The RSV replication curve showed that RSV replication approximated its peak around 72 h post infection (hpi). RSV replication was comparable between both the adult and pediatric HAE cultures (Supplemental Figure S1). Light microscopy of RSV-infected HAE at 72 hpi showed no cilia destruction or the presence of a cytopathogenic effect (CPE) [23]. However, compared to controls, SEM imaging of RSV-infected HAE cultures at 72 hpi showed culture damage in a disrupted, patchy pattern and areas with loss of ciliated cells. This appeared to have increased at 120 hpi (Figure 2), although also in the control, regions with damaged ciliated cells were detected. To study the effect of NETs in the setting of RSV infection, but before severe loss of cilia due to RSV infection, NETs (next paragraph) were added to cultures at 48 h post infection. Subsequent read-outs were performed at 72 h post infection.

### 4.2. NET Production

Production of NETs by PMA-stimulated neutrophils was confirmed with immunofluorescence confocal imaging that visualized the presence of neutrophil elastase and citrullinated histones on extracellular DNA (Supplemental Figure S2). For quantification of NETs, we determined the concentration of dsDNA in the supernatants: dsDNA concentration in NET supernatant ranged from 123–327 ng/mL, as compared to 8.78–14.7 ng/mL in the supernatant of the unstimulated neutrophils. The addition of NETs supernatant did not interfere with viral RSV replication in HAE (Supplemental Figure S1).

### 4.3. NETs Did Not Induce Injury to (RSV-Infected) HAE Cultures 

**Figure 3:**
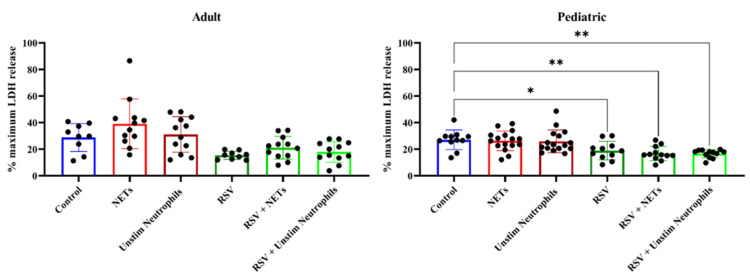
Percent LDH release by HAE cell cultures after 24 h of exposure to experimental compounds. Percent LDH release is calculated as a relative percentage against the maximum of LDH release. Although a one-way ANOVA for multiple comparisons indicated that there were differences between the experimental groups for both adult cell cultures (F 6.57, *p* <0.0001) and pediatric cell cultures (F 6.57, *p* < 0.0001), post hoc testing showed only in the pediatric donor a decreased LDH release in RSV-infected cultures compared to controls (Dunnett’s multiple comparison for control versus RSV adjusted *p* = 0.03, RSV + NETs adjusted *p* < 0.01, RSV + unstimulated neutrophil supernatant adjusted *p* < 0.01). * *p* < 0.05, ** *p* < 0.01.

**Figure 4:**
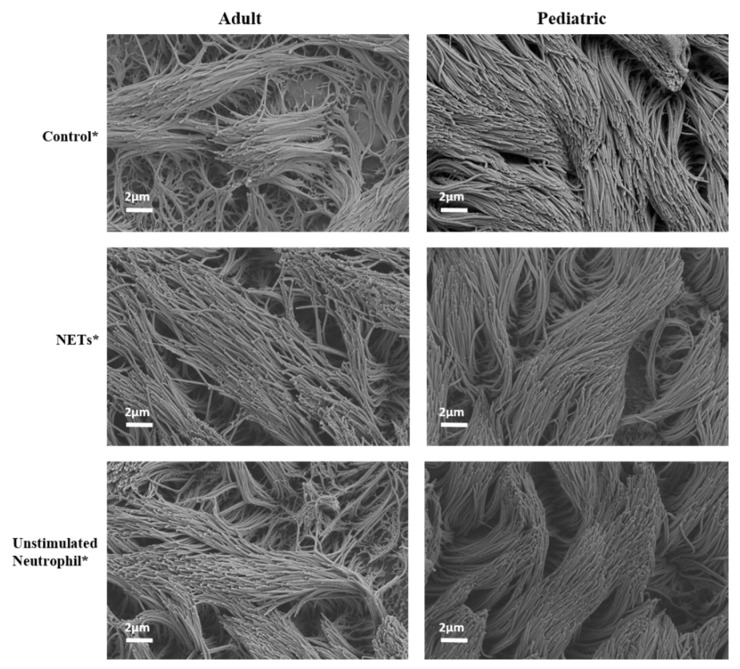
Scanning electron microscopy (SEM) pictures of HAE cultures for the different experimental groups (control, NETs and unstimulated neutrophil supernatant). Legend: SEM pictures taken at 5.00 k× magnification (Zeiss). * HAE culture exposed for 24 h to PBS only (control), NETs (NET supernatant) and unstimulated neutrophils (supernatant of unstimulated neutrophils).

Exposure of both the adult and pediatric HAE cultures to supernatant containing NETs did not lead to increased release of LDH, a marker of cytotoxicity, as compared to supernatant of unstimulated neutrophils or PBS (Figure 3). Similar findings were observed in the HAE cultures with active RSV infection. Both the adult and pediatric HAE cultures showed significant differences in LDH release between the experimental groups (ANOVA, *p* < 0.0001); however, only the pediatric HAE cultures showed a reduction in LDH release in all three RSV-infected HAE experimental conditions as compared to controls via post hoc Dunnett’s tests (Figure 3). Thus, this decreased LDH release in RSV-infected pediatric cultures was unrelated to the exposure to NETs. Consistent with the findings for LDH release, we did not observe any destruction of the ciliated cells by electron microscopy in either the adult or pediatric cultures exposed to NETs (Figure 4).

### 4.4. RSV Infection, but Not NETs, Caused Increased Pro-Inflammatory Cytokine Release from HAE Cultures 

**Figure 5:**
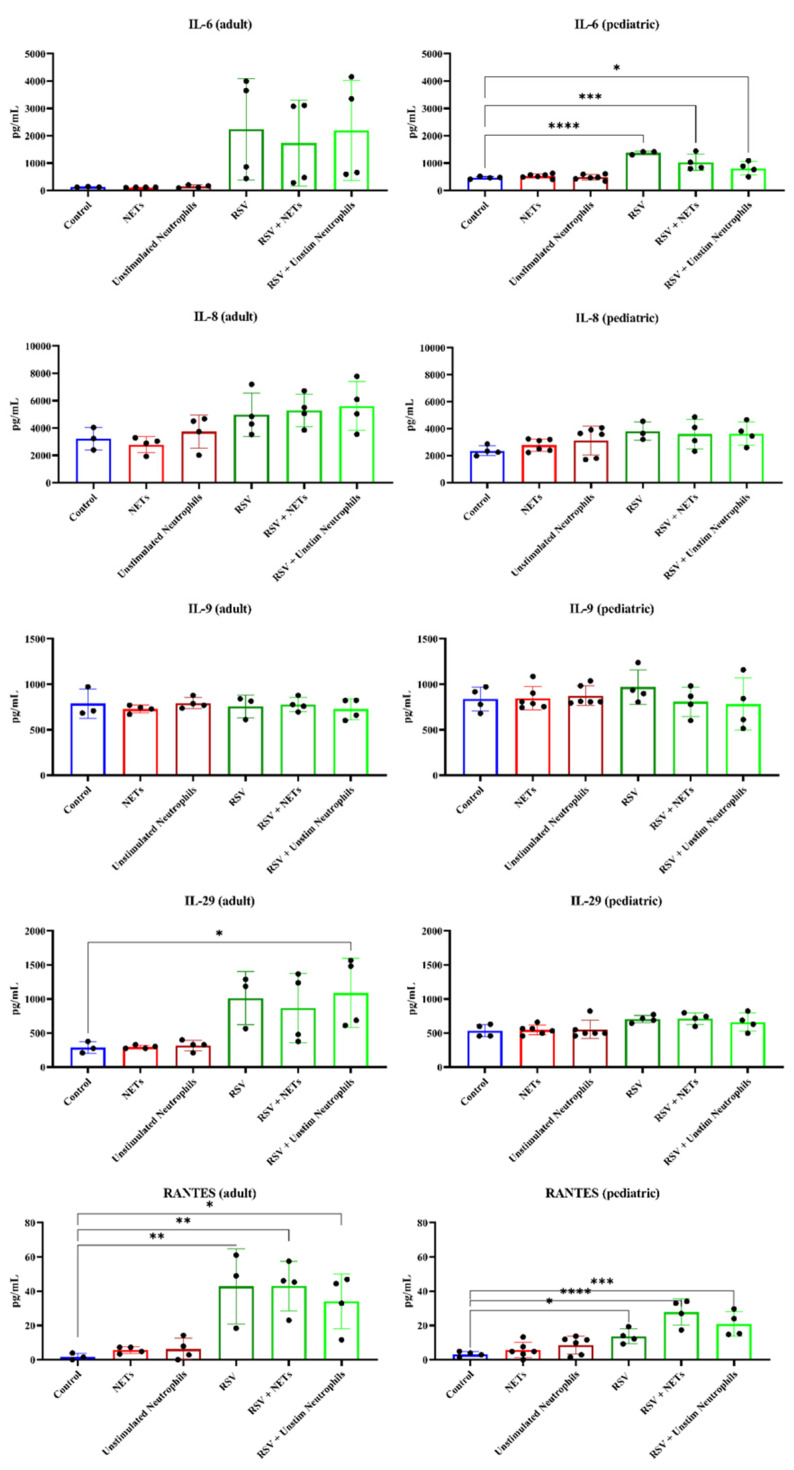
Cytokine release by HAE cultures for the different experimental groups. Each Luminex datapoint represents pooled samples of n = 3 inserts. Asterisks indicate significant outcomes for a Dunnett’s multiple comparisons test to compare the cytokine release in the different experimental groups compared to control cultures (* *p* < 0.05, ** *p* < 0.01, *** *p* < 0.001, **** *p* < 0.0001), *Y*-axis in pg/mL. Dunnett’s multiple comparison test for IL-6 release in the RSV-infected cell cultures compared to controls (PBS only): mean difference in pg/mL 909.7, adj *p* < 0.0001, RSV + NETs 558.2, adj *p* < 0.01, RSV + Unstim Neutrophils 341.5, adj *p* < 0.05. The release of RANTES in the adult donor cell cultures compared to controls was in pg/mL RSV 41.02, adj *p* < 0.01, RSV + NETs 41.17, adj *p* < 0.01, RSV + Unstim Neutrophils 32.19, adj *p* < 0.05. For the pediatric donor this was RSV 10.42, adj *p* < 0.05, RSV + NETs 24.59, adj *p* < 0.0001, RSV + Unstim Neutrophils 17.64, adj *p* < 0.001.

RSV infection of both adult and pediatric HAE cultures led to an increase in the release of several pro-inflammatory cytokines, including IL-6, IL-8, IL-29 and RANTES (Figure 5). Except for IL-29, which showed a prominent increase only in the RSV-infected adult HAE, the pattern of these cytokine changes was mostly similar for both adult and pediatric HAE cultures. However, no (additional or synergistic) effects on pro-inflammatory cytokine release in HAE cultures were found upon exposure to the supernatant containing NETs, as compared to the supernatant of unstimulated neutrophils (Figure 5). 

### 4.5. Age-Related Differential Gene Expression to RSV Infection in HAE Cultures

**Figure 6:**
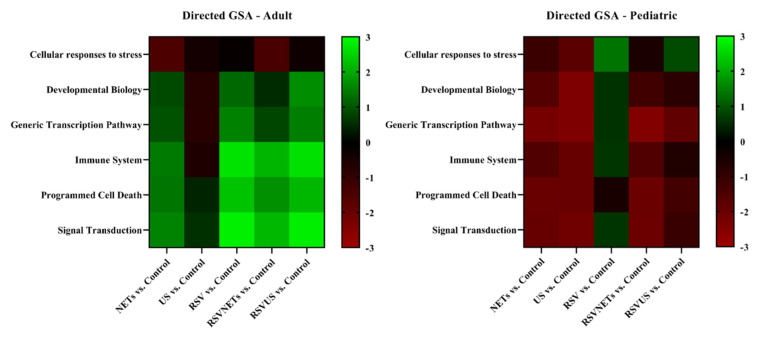
Heat maps for directed gene set analysis (GSA) for the comparison of each experimental group against the controls. GSA summarizes the change in expression within each defined gene set (pathway) for each comparison. Left: adult, right: pediatric. Light green indicates upregulation, red indicates downregulation compared to the baseline. Genes related to the pathways’ immune system and programmed cell death are presented in Supplemental Table S1.

Finally, to be able to detect more upstream changes in common cellular response pathways, we explored gene expression profiles in the HAE cultures exposed to RSV and NETs. In the analysis of the adult HAE cultures all samples were included, but three pediatric HAE cultures (one exposed to NETs and two exposed to unstimulated neutrophil supernatant) were excluded from the final analysis due to technical errors. Exposure to NETs did not cause a statistically significant up- or downregulation of gene sets related to apoptosis or immune system activation as studied here in both adult and pediatric HAE cultures. However, we did observe differences in the differential gene expression responses to RSV infection between the adult and pediatric HAE cultures, as studied via directed gene set analysis. Namely, as compared to the uninfected control, RSV infection resulted in a statistically significant upregulation of several genes involved in basic immunological, signal transduction and programmed cell death pathways for the adult HAE (Figure 6).

Genes most strongly upregulated were *TNFSF10* (*TNF superfamily member 10*, adj *p* < 0.01), *FAS* (*FAS cell surface death receptor*, adj *p* < 0.05), *CASP1* (*caspase 1*, adj *p* < 0.01), *MYD88* (*myeloid differentiation protein 88*, adj *p* < 0.01), and *RIPK1* (*receptor-interacting serine/threonine-protein kinase 1*, adj *p* < 0.05). Both *TNFSF10* and *FAS* are involved in extrinsic apoptosis. In contrast, in the pediatric donor HAE, none of these genes were significantly up- or downregulated. Instead, in these cells there was a downregulation of genes *TNFRSF10B* (adj *p* < 0.01), *BAX* (*Bcl2-associated X-protein*, adj *p* < 0.05), *GADD45A* (*growth arrest and DNA damage-inducible 45 protein*, adj *p* < 0.05), and *TP53BP2* (*Tumor Protein P53 Binding Protein 2*, adj *p* < 0.05) in all experimental groups except for the RSV-only compared to controls. Lists of the five genes most up- or downregulated per experimental comparison are presented in Supplemental Table S4.

## 5. Discussion

This study aimed to determine whether NETs induce epithelial injury or pro-inflammatory responses in adult and pediatric bronchial HAE cultures in the setting of RSV infection. In the current study set-up, 24 h exposure to NETs did not induce a cytotoxic pro-inflammatory response or aggravate RSV-induced cytotoxicity or inflammation in HAE cultures. 

Previous literature that studied neutrophilic influx in the airways in RSV-LRTI showed that the co-culturing and transepithelial migration of neutrophils in RSV-infected HAE cultures caused epithelial injury with loss of cilia, decreased cilia beating and cell detachment [10,28]. Neutrophils can respond to RSV infection via the release of NETs [11], and involvement of NETs in the emergence of airway mucus plugs in RSV-LRTI has been implicated [11,14,26]. However, the direct effects of NETs on the airway epithelium in RSV-LRTI remain to be elucidated. In contrast to previous literature, we did not observe a direct cytotoxic or inflammatory effect of NETs on the bronchial airway epithelium [18]. This could possibly be explained by the low concentration of NETs added in this study as compared to previous studies [15,16,18]. Literature reporting on the effect of NETs for the airway epithelium is heterogeneous in the reported methods for neutrophil stimulation as well as the cell culture models used [15,16,17,18]. Monolayer cultures are submerged by design, and this allows for the apical addition of large volumes of NET-containing fluid to the cell cultures. However, a different approach is necessary for the experimental exposure of HAE cultures, as the apical side of HAE cultures is air exposed. Therefore, we considered that the addition of large amounts of fluids to the HAE cultures for a prolonged period of time could result in cell drowning and cell detachment. Limited by the maximum suspension volume that may be added to the HAE cultures, without risking cell detachment, the concentration of NETs in the added suspension must therefore be higher, in order to expose the HAE cultures to comparable concentrations as previously reported in the literature. In addition, the translation of the in vivo to the in vitro exposure of the airway epithelium is challenging. For example: in children with severe RSV-LRTI, the DNA content in bronchoalveolar lavage fluids (BALFs) ranges from 60–611 μg/mL as compared to 44–96 μg/mL in healthy infants [37], and this cannot easily be mimicked in cell culture models. 

A study by Hudock et al. previously reported inflammatory responses of HAE cultures after exposure to NETs [18]. However, these NETs were obtained by stimulating neutrophils with high dosages of phorbol myristate acetate (PMA, 500 nM), followed by the scraping of neutrophils from the culture plate and vigorous mixing [18]. In our study, we chose to use a ten-fold lower dosage of PMA for neutrophil stimulation as compared to Hudock et al. [18], using PMA dosages for NET harvesting comparable to the literature [15,16,17,26]. At the dose of 50 nM, PMA induces NET formation rather than other forms of neutrophilic death [15,38,39], and we have previously shown that NETs collected following our method induce a significant increase in airway mucus viscoelasticity [26]. However, in our study, NETs did not cause or aggravate RSV-induced airway epithelial inflammation or injury. Thus, we are unable to draw any conclusions about the effect of NETs for the airway epithelium. Interestingly, previous literature reported a dose-dependent deleterious effect of NETs on A549 (human adenocarcinoma alveolar basal epithelial) cells [15], but an anti-inflammatory effect of NETs has also been described in the context of gout [40]. 

Severe RSV-LRTI mostly affects young children and therefore well-differentiated HAE cultures from primary cells of age-related donors may theoretically allow for the detection of age-related differences in the RSV–host airway epithelium interaction. In this study, RSV infection and replication caused a pro-inflammatory response in HAE cultures via the release of RANTES and IL-6. This adds to previous in vitro and in vivo data that reported the release of IL-6 and RANTES (CCL-5), a leukocyte attractant by RSV-infected airway epithelium [41,42,43]. The cell culture injury in RSV-infected adult HAE cultures at 72 hpi was not reflected by increased LDH release from these adult cell cultures at this time point. Instead, RSV-infected pediatric cultures indicated a decreased LDH release compared to uninfected controls. The cell sloughing and destruction of the ciliated cells is a common characteristic reported by histopathological studies for RSV in vivo. To further study cell injury-related and cell death-related mechanisms by RSV, we used a multiplex RNA panel to study programmed cell death and related pathways. Interestingly, we observed an upregulation of several immune and programmed cell death-related genes in the adult, but not in the pediatric RSV-infected HAE cultures. 

Programmed cell death, or apoptosis, is a tightly regulated and energy-dependent form of cell death [44] that can occur in airway epithelial cells upon viral infection and replication [45,46]. In RSV-LRTI, apoptosis of airway epithelial cells has been proposed as a host defense mechanism to limit viral spreading in the airways during later stages of RSV infection [7,23,29,47]. The upregulation of the death receptor pathway genes *TSNF10* and *FAS* in the adult HAE cultures falls into context with the previous findings of RSV-induced sensitization of airway epithelial cells for TRAIL (tumor necrosis factor-related apoptosis-inducing ligand)-induced external apoptosis pathways by effector immune cells such as leukocytes [47,48,49]. Elevated levels of soluble TRAIL in bronchoalveolar lavage fluid (BALF) of invasively ventilated children for severe RSV-LRTI was shown previously [49]. However, the upregulation of apoptosis pathways was not observed in pediatric HAE cultures. Future studies should include HAE cultures obtained from multiple pediatric and adult donors to further clarify the reported differences in LDH release and pro-apoptotic responses between RSV-infected HAE cultures. Several limitations with respect to the findings presented in this study should be considered. As discussed above, all cultures originated from two human donors and it is thus possible that differences in the study outcomes for adult and pediatric cultures may be the result of donor-related, rather than age-related, factors. Although the use of HAE culturing is more relevant for the in vivo situation compared to conventional monolayer cell studies, the culturing of HAE is laborious, requires specific expertise and can be challenged by the limited availability of primary cell donors. 

## 6. Conclusions

In conclusion, in our study NETs did not cause or aggravate RSV-induced airway injury or inflammation in human airway epithelial (HAE) cultures in vitro. Future research should further investigate the potential deleterious effects of NETs in the setting of RSV-LRTI, carefully choosing the method of neutrophil stimulation as well as the cell culture model. 

## Data Availability

Data may be shared upon reasonable request.

## Supplementary Materials

The following supporting information can be downloaded at: https://www.mdpi.com/article/10.3390/cells11050785/s1, Figure S1: RSV replication in HAE cultures by RT-qPCR, Figure S2: Confocal images of unstimulated neutrophils and NETs, Table S1: List of genes included in the CodeSet analysis, Table S2: Cytokine release for the different experimental groups, Table S3: Directed differential expression (gene set analysis), Table S4: The five most up- or downregulated genes per comparison.
