# Supplementary material for: Neutrophil Extracellular Traps Do Not Induce Injury and Inflammation in Well-Differentiated RSV-Infected Airway Epithelium
Source: Cells. 2022 Feb 24;11(5):785. doi: 10.3390/cells11050785 (PMC8909397; doi:10.3390/cells11050785)
Supplement: Supplementary file 1 [file cells-11-00785-s001.zip › cells-1585098-supplementary.pdf]

Supplemental Data

Supplemental Table S1: List of Genes included in the CodeSet analysis
Legend: # number in the gene set. I = gene involved in immune system (NanoString); A = gene involved in regulated cell death or apoptosis (NanoString).

Supplemental Table S2: Cytokine release for the different experimental groups
Data are presented as mean (SD) in pg/ml. One-way ANOVA for multiple comparisons F, (p).

Supplemental Table S3: Directed differential expression (Gene set analysis)
Legend: NETs: neutrophil extracellular traps, US: unstimulated neutrophil supernatant, RSV: respiratory syncytial virus. Ped*: pediatric.

Supplemental Table S4: The five most up- or downregulated genes per comparison are displayed, or more in the case more were significantly up- or downregulated. Legend * adjusted p <0.05 ** adjusted p <0.01, *** adjusted p <0.001. NETs: neutrophil extracellular traps, US: unstimulated neutrophil supernatant, RSV: respiratory syncytial virus

Supplemental Figure S1: RSV replication in HAE cultures by RT-qPCR in adult and pediatric HAE cultures.

Supplemental Figure S2: Confocal Images of Unstimulated Neutrophils and NETs
Legend: Upper row: neutrophils and NETs stained for Citrullinated Histone 3 (yellow), lower row neutrophil elastase (NE, magenta), and DNA (DAPI, blue).

Supplemental Table S1: List of Genes included in the CodeSet analysis
Legend: # number in the gene set. I = gene involved in immune system (NanoString); A = gene involved in regulated cell death or apoptosis (NanoString).

#	*Gene*	I	A	#		I	A	#		I	A
1	ABCF1 (HK)			39	CASP8	x	x	77	RPLP0 (HK)		
2	ABL1	X		40	CASP9			78	TNF	X	
3	AIFM1			41	CD27	X		79	TNFRSF10A		X
4	AKT1	X	X	42	CD40	X		80	TNFRSF10B		X
5	APAF1	X	X	43	CD40LG	X		81	TNFRSF11B	X	
6	BAD		X	44	CD70	X		82	TNFRSF1A	X	
7	BAG1			45	CFLAR		X	83	TNFRSF1B	X	
8	BAG3			46	CIDEA			84	TNFRSF21		
9	BAK1			47	CIDEB			85	TNFRSF25	X	
10	BAX		X	48	CRADD			86	TNFRSF9	X	
11	BBC3		X	49	CYCS		X	87	TNFSF10		X
12	BCL10	X		50	DAPK1		X	88	TNFSF8	X	
13	BCL2	X	X	51	DAXX			89	TP53	X	X
14	BCL2A1			52	DFFA		X	90	TP53AIP1		
15	BCL2L1	X	X	53	DIABLO		X	91	TP53BP2		X
16	BCL2L10			54	FADD	X	X	92	TP73		X
17	BCL2L11		X	55	FAS			93	TRADD		X
18	BCL2L2			56	FASLG	X	X	94	TRAF2	X	X
19	BFAR			57	GADD45A			95	TRAF3	X	
20	BID		X	58	GUSB (HK)			96	XIAP		X
21	BIK			59	HPRT1 (HK)			97	MYD88	X	
22	BIRC2	X	X	60	HRK			98	RIPK1	X	X
23	BIRC3		X	61	IGF1R						
24	BIRC5	X		62	IL10	X					
25	BIRC6			63	LDHA (HK)						
26	BNIP2			64	LTA	X					
27	BNIP3			65	LTBR	X					
28	BNIP3L			66	MCL1	X					
29	BRAF	X		67	NAIP						
30	CASP1	X		68	NFKB1	X					
31	CASP10	X		69	NOD1	X					
32	CASP14			70	NOL3						
33	CASP2	X		71	PARP1						
34	CASP3	X	X	72	PIDD1						
35	CASP4	X		73	POLR1B (HK); excluded in final analysis						
36	CASP5			74	PRF1						
37	CASP6		X	75	PYCARD	X					
38	CASP7		X	76	RIPK2	X					

Supplemental Table S2: Cytokine release for the different experimental groups

Data are presented as mean (SD) in pg/ml. One-way ANOVA for multiple comparisons F, (p).

Pooled

Mean (SD)	Control	NETs	Unstimulated Neutrophils	RSV	RSV + NETs	RSV +Unst. Neutrophils	ANOVA
**IL-6**	324.5 (182.7)	363.3 (202.2)	349.9 (192.3)	1869 (1383)	1381 (1112)	1500 (1418)	F 4.884 p<0.01
**IL-8**	2729 (709.0)	2781 (479.0)	3364 (1111)	4468 (1340)	4440 (1390)	4622 (1673)	F 4.611 p <0.01
**IL-9**	816.2 (134.4)	800.0 (117.4)	841.4 (97.0)	877.3 (189.4)	792.7 (117.5)	754.6 (204.1)	F 0.6636 ns
**IL-29**	429.6 (155.2)	446.2 (139.8)	458.9 (164.5)	836.6 (282.3)	789.1 (347.6)	872.9 (412.4)	F 5.383 p<0.001
**RANTES**	2.631 (1.718)	5.652 (3.494)	7.637 (5.396)	26.16 (20.30)	35.41 (13.40)	27.44 (13.50)	F 12.68 p<0.001

Adult

Mean (SD)	Control	NETs	Unstimulated Neutrophils	RSV	RSV + NETs	RSV +Unst. Neutrophils	ANOVA
**IL-6**	131.9 (9.5)	116.5 (8.1)	146.8 (51.1)	2237 (1844)	1736 (1571)	2189 (1834)	2.68 ns
**IL-8**	3223 (816.5)	2784 (591.7)	3735 (1217)	4968 (1582)	5280 (1180)	5613 (1783)	3.21 P<0.05
**IL-9**	787.7 (160.5)	728.7 (42.0)	792.9 (60.4)	755.4 (125.5)	777.4 (75.74)	726.8 (113.1)	0.34 ns
**IL-29**	287.3 (84.5)	296.1 (26.0)	316.9 (78.8)	1013 (391.2)	865.1 (509.1)	1087 (506.4)	4.55 P<0.01
**RANTES**	1.79 (1.97)	5.66 (1.88)	6.25 (6.25)	42.8 (21.9)	43.0 (14.4)	34.0 (16.1)	8.85 P<0.001

Pediatric

Mean (SD)	Control	NETs	Unstimulated Neutrophils	RSV	RSV + NETs	RSV +Unst. Neutrophils	ANOVA
**IL-6**	468.9 (42.5)	527.7 (78.0)	485.3 (100.0)	1379 (62.6)	1027 (293.7)	810.4 (245.9)	19.8 P<0.0001
**IL-8**	2357 (363.9)	2779 (450.5)	3117 (1072)	3801 (683.8)	3599 (1109)	3631 (856.3)	1.91 ns
**IL-9**	837.5 (132.5)	847.5 (130.2)	873.8 (107.7)	968.8 (187.7)	807.9 (160.7)	782.4 (287.0)	0.60 ns
**IL-29**	536.4 (89.5)	546.3 (68.5)	553.5 (134.6)	704.2 (53.0)	713.2 (84.0)	658.7 (134.9)	3.02 p<0.05
**RANTES**	3.26 (1.45)	5.65 (4.46)	8.56 (5.14)	13.7 (4.25)	27.9 (7.67)	20.9 (7.20)	13.7 P<0.0001

Supplemental Table S3: Directed differential expression (Gene set analysis)
Legend: NETs: neutrophil extracellular traps, US: unstimulated neutrophil supernatant, RSV: respiratory syncytial virus. Ped*: pediatric.

Directed Treatment: differential expression	expression in NETS vs. baseline of Control		expression in US vs. baseline of Control		expression in RSV vs. baseline of Control		expression in RSVNETS vs. baseline of Control		expression in RSVUS vs. baseline of Control	
	adult	ped*	adult	ped*	adult	ped*	adult	ped*	adult	ped*
Cellular responses to stress	-1,428	-1,117	-0.395	-1706	-<br>0.113	1,355	-1,377	-0.506	-0.289	0.875
Developmental Biology	0.869	-1,584	-0.733	-2.4	1,218	0.574	0.487	-1212	1,649	-0.804
Generic Transcription Pathway	0.962	-2,234	-0.777	-2393	1.51	0.582	0.814	-2449	1,475	-1833
Immune System	1,432	-1,509	-0.592	-1925	2,663	0.613	2,115	-1,512	2,639	-0.62
Programmed Cell Death	1,368	-1,965	0.423	-1952	2,292	-0.473	1,685	-2,015	2,151	-1.26
Signal Transduction	1,547	-1.92	0.561	-2121	2.82	0.608	2,159	-2,027	2,796	-1067

Supplemental Table S4: **The five most up- or downregulated genes per comparison** are displayed, or more in the case more were significantly up- or downregulated. Legend * adjusted p <0.05 ** adjusted p <0.01, *** adjusted p <0.001. NETs: neutrophil extracellular traps, US: unstimulated neutrophil supernatant, RSV: respiratory syncytial virus

	Gene	Log 2 fold change (confidence interval)	Adjusted P
**Adult**			
Control vs. RSV	TNFSF10	1.42 (1.12 – 2.68)	6.34x10^-7***
	MYD88	1.04 (0.122 – 0.801)	1.56x10^-6***
	CASP1	0.927 (0.133 – 0.667)	3.24x10_7***
	FAS	0.873 (0.536 – 1.21)	3.43x10-5 ***
	RIPK1	0.527 (0.271 - 0.783)	0.0298*
	CASP7	0.311 (0.157 – 0.464)	0.0306*
Control vs. NETs	CASP10	0.423 (0.108 – 0.211)	0.205
	BIRC2	0.375 (0.165 – 0.587)	0.296
	CFLAR	0.199 (0.0787 – 0.318)	0.34
	CASP7	0.214 (0.0809 – 0.347)	0.34
	BNIP3	0.332 (0.0895 – 0.575)	0.692
Control vs. US	MCL1	-0.32 (-0.503 – -0.137)	0.54
	GADD45A	-0.588(-0.943 – -0.233)	0.54
	FAS	0.387 (0.0951 – 0.679)	1
	BCL10	-0.217 (-0.396 – -0.0386)	1
	APAF1	-0.198 (-0.383 – -0.0386)	1
Control vs. RSV +NETs	MYD88	0.718 (0.512 – 0.925)	0.000152***
	TNSF10	0.845 (0583 – 1.11)	0.000241***
	CASP1	0.553 (0.328 – 0.779)	0.00713**
	FAS	0.552 (0.23 – 0.814)	0.144
	RIPK1	0.377 (0.156 – 0.599)	0.172
Control vs. RSV + US	MYD88	0.874(0.667 – 1.08)	5.37x10^-6***
	TNSF10	1.01 (0.753 – 1.28)	1.2 x 10^-5***
	CASP1	0.638 (0.413 – 0.864)	0.0011**
	FAS	0.638 (0.413 – 0.864)	0.0229*
	RIPK1	0.466 (0.113 – 0.245)	0.024*
**Pediatric**			
Control vs. RSV	PARP1	0.374 (0.165 – 0.584)	0.655
	TNFSRF10B	-0.431 (-0.705 – -0.157)	0.867
	BAG1	0.4 (0.0622 – 0.738)	1
	AIFM1	0.264 (0.0286 – 0.499)	1
	CASP9	0.327 (0.0343 – 0.619)	1
Control vs. NETs	TNFRSF10B	-0.703 (-0.948 – -0.458)	0.00376**
	BAX	-0.5 (-0.682 – -0.318)	0.00376**
	GADD45A	-0.879 (-1.26 – -0.499)	0.0186*
	MCL1	-0.274 (-0.432 – -0.115)	0.216
	TP53BP2	-0.449 (-0.727 – -0.172)	0.254
Control vs. US	TNFSRF10B	-0.703 (-0.959 – -0.446)	0.00774**
	GADD45A	-1.02 (-1.42 – -0.623)	0.00855**
	BAX	-0.44 (-0.634 – -0.254)	0.017*
	BRAF	-0.458 (-0.685 – -0.231)	0.0487*
	CFLAR	-0.345 (-0.518 – -0.173)	0.0487*
Control vs. RSV +NETs	TNFSRF10B	-0.764 (-1 – -0.527)	0.00091***
	GADD45A	-1 (-1,37 – -0.633)	0.00425**
	BAX	-0.403 (-0.579 – -0.226)	0.0213*
	TP53BP2	-0.551 (-0.82 – -0.282)	0.0479*
	BCL10	-0.338 (-0.536 – -0.141)	0.186
Control vs. RSV + US	TNFRSF10B	-0.72 (-0.957 – -0.483)	0.00207**
	GADD45	-0.964 (-1.33 – -0.597)	0.00664**
	TNSF10	0.798 (0.394 – 1.2)	0.0924
	PARP1	0.32 (0.139 – 0.502)	0.163
	TP53BP2	-0.467 (-0.736 – -0.199)	0.163

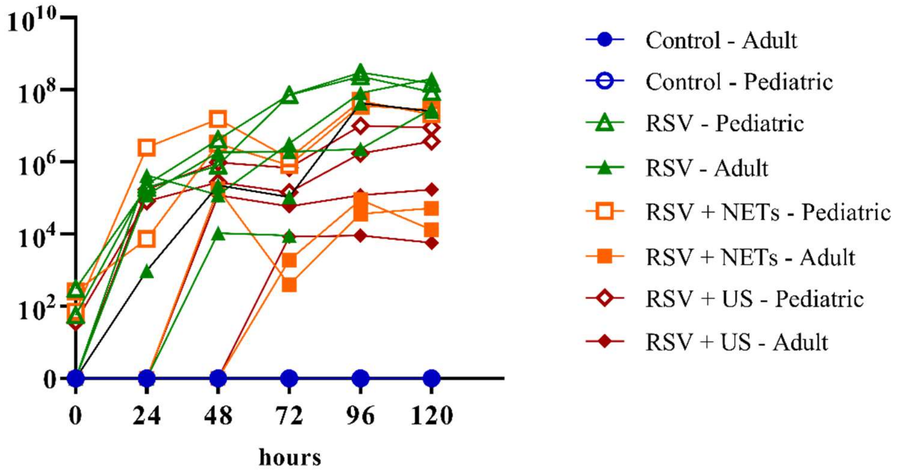

Supplemental Figure S1: RSV replication in HAE cultures by RT-qPCR in adult and pediatric HAE cultures. Control: uninfected control, RSV: RSV infection only, RSV +NETs: RSV infected and NETs supernatant added at 48 hours, RSV + US: RSV infected and unstimulated neutrophil supernatant added at T48 hours. All RSV infected HAE cultures were infected at T0.

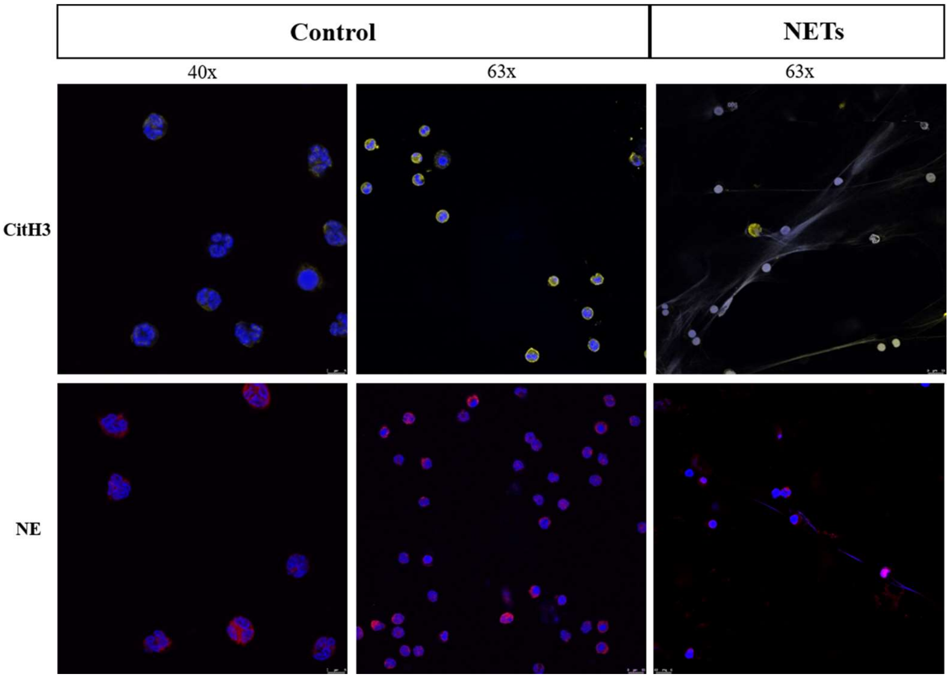

Supplemental Figure S2: Confocal Images of Unstimulated Neutrophils and NETs
Legend: Upper row: neutrophils and NETs stained for Citrullinated Histone 3 (yellow), lower row neutrophil elastase (NE, magenta), and DNA (DAPI, blue).
